# Aloe-emodin induces apoptosis in human oral squamous cell carcinoma SCC15 cells

**DOI:** 10.1186/s12906-018-2353-z

**Published:** 2018-11-07

**Authors:** Qihong Li, Jun Wen, Kaitao Yu, Yao Shu, Wulin He, Hongxing Chu, Bin Zhang, Cheng Ge

**Affiliations:** 1 Department of Stomatology, 307 Hospital, PLA, 8 Dongda Street, Beijing, 100071 China; 2 Stomatological Hospital, Southern Medical University, No. 366, South Jiangnan Avenue, Guangzhou, 510280 China; 3 Department of Stomatology, Chinese PLA General Hospital, 28 Fuxing Road, Beijing, 100853 China

**Keywords:** Aloe-emodin, Anthraquinone, Apoptosis, Oral squamous cell carcinoma, SCC15 cells

## Abstract

**Background:**

Oral and pharyngeal cancer is the most common malignant human cancers. Chemotherapy is an effective approach for anti-oral cancer therapy, while the drug tolerance and resistance remain a problem for oral cancer patients. Aloe-emodin, rhein and physcion are classified as anthraquinones, which are the main pharmacodynamic ingredients of *Rheum undulatum L.*. This study was undertaken to investigate whether aloe-emodin, rhein and physcion show inhibiting growth and inducing apoptosis in oral squamous cell carcinoma SCC15 cells. We found that aloe-emodin show inhibiting growth and inducing apoptosis in oral squamous cell carcinoma SCC15 cells, we also investigated the underlying mechanisms of apoptosis induced by aloe-emodin.

**Methods:**

Thiazolyl blue tetrazolium bromide (MTT) test was used to detect cell proliferation. Cell apoptosis was detected by flow cytometry. We also used western blot analysis to detect the potential mechanisms of apoptosis.

**Results:**

Aloe-emodin, rhein and physcion inhibit the proliferation of SCC15 cells and the order of inhibition level are aloe-emodin > Rhein > Physcion, the half maximal inhibitory concentrations (IC_50_) value of aloe-emodin was 60.90 μM at 48 h of treatment. Aloe-emodin treatment resulted in a time- and dose-dependent decrease in cell viability and increased the apoptotic cell ratio. The results of western blotting showed the expression levels of caspase-9 and caspase-3 proteins increased following aloe-emodin treatment.

**Conclusions:**

Our results revealed that aloe-emodin treatment could inhibit cell viability of SCC15 cells and the potential mechanism of inhibition might be through the induction of apoptosis by regulation of the expression levels of caspase-9 and caspase-3. This indicates that aloe-emodin may be a good agent for anti-oral cancer drug exploring.

## Background

Oral and pharyngeal cancer is the sixth most common malignant human cancers worldwide [1]. Despite advancements in cancer treatment, the 5-year survival rate of oral cancer patient is less than 50% [2, 3]. Chemotherapy is an effective and useful approach for anti-oral cancer therapy, meanwhile, the drug tolerance and resistance remains an issue for oral cancer patients. Thus, a better and safe chemical molecular for this disease therapy is to develop.

**Fig. 1:**
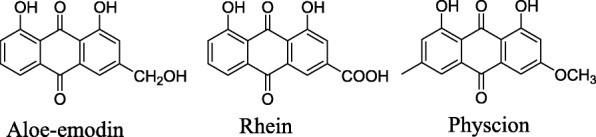
Structures of aloe-emodin, rhein and physcion

With the aim of developing novel anti-oral cancer drugs, we devoted our attention to natural compounds that have been used to treat a variety of cancer diserases. Aloe-emodin, rhein and physcion (Fig. 1) derived from *Rheum undulatum L.* have potent biological effects. Despite much evidence suggesting that aloe-emodin, rhein and physcion show anticancer activity in many cancer cell lines, such as against human hepatoblastoma cell, colorectal cancer cells and human melanoma cells [4–6], there is not enough information to show that these compounds. Against the human oral cancer cells.

Therefore, in this study, we examined the effect of aloe-emodin, rhein and physcion on the growth of human oral squamous cell carcinoma cell line SCC15. The results demonstrated that aloe-emodin, rhein and physcion inhibit the proliferation of SCC15 cells and the order of inhibition level is aloe-emodin > rhein > physcion. Our results showed that aloe-emodin could induce SCC15 cells apoptosis, moreover, the expression levels of caspase-9 and caspase-3 increased suggesting that the potential mechanism of aloe-emodin induces apoptosis might by regulating the caspases in SCC15 cells.

## Methods

### Reagents and chemicals

Dulbecco’s modified Eagle’s medium (DMEM), phosphate buffered saline (PBS), and fetal bovine serum (FBS) were purchased from Gibco (Thermo Fisher Scientific, NY, USA). 96-Well plates were purchased from Corning Costar (Corning Inc., NY, USA). Aloe-emodin (Cat No. 110795–201710), rhein (Cat No. 110757–201607), physcion (Cat No. 110758–201616) (> 98% pure, free of endotoxin) were purchased from National Institutes for Food and Drug Control (Beijing, China), which were dissolved in DMSO and passed through a 0.22 μm filter (Pall Life Sciences, MI, USA) for sterilization and diluted with culture medium to final concentrations before treatment. In all experiments, the final DMSO concentration did not exceed 1‰ (*v*/v), so as not to affect cell growth. Thiazolyl blue tetrazolium bromide (MTT) was purchased from Sigma Sigma-Aldrich Co. (St. Louis, MO, USA). Caspase-3 (8G10) Rabbit mAb (Cat No. 9665), Caspase-9 antibody (human specific) (Cat No. 9502), β-actin (13E5) Rabbit mAb (Cat No. 4970), anti-rabbit IgG, HRP-linked antibody (Cat No.7074) were obtained from Cell Signaling Technology (Danvers MA USA).

### Cell culture and chemical treatment

Human oral squamous cell carcinoma cell line SCC15 was donated by Zhang Xin-yan, professor of Capital medical university school of stomatology (Beijing, China), which was obtained from the American Type Culture Collection (Manassas VA) and stored in our laboratory. The cells were cultured in DMEM containing with 10% FBS, 100 U/ml penicillin, 100 μg/ml streptomycin and incubated at 37 °C in a humidified atmosphere containing 5% CO_2_.

### Cell viability assay

Cell viability was evaluated by MTT assay [7]. SCC15 cells were seeded at 1 × 10^4^ cells/ml in 96-well plates and cultured for 24 h. After treatment with various concentrations of the test compounds for 24 h, 48 h or 72 h, 0.5 mg/ml MTT was added and incubated with cells for 4 h at 37 °C under 5% CO_2_. The medium was removed and DMSO (150 μl) was added to each well. The optical density (OD) was measured at 492 nm by a Microplate Reader (Multiskan MK3, Thermo). The percentage of cell viability was calculated according to the following formula: (OD value of the control cells – OD value of the treated cells) / OD value of the control cells × 100%. By definition, the viability of the control cells from the untreated cultures was defined as 100%. The IC_50_ value was calculated by Graph Pad Prism 6.0.

### Apoptosis analysis by flow cytometry

Apoptosis was measured using flow cytometry to quantify the levels of detectable phosphatidylserine on the outer membrane of apoptotic cells [8]. Aloe-emodin induces apoptosis of the cells was measured using an Annexin V- Fluorescein isothiocyanate (FITC)/ propidium iodide (PI) apoptosis detection kit (Solarbio life Sciences, Beijing, China) according to the manufacturer’s protocol. In brief, SCC15 cells treated with or without aloe-emodin for 24 h or 48 h were collected by trypsinization and washed twice with cold PBS. After centrifugation, the cell pellets were resuspended in a 500 μl binding buffer solution. Then, 5 μl of Annexin V-FITC and 5 μl of PI solutions were added and the mixtures were further incubated in the dark for 30 min at room temperature. The Annexin V-FITC and PI fluorescence of cultured cells were analyzed by flow cytometry (Becton Dickinson FACSCalibur, USA).

### Western blot assay

SCC15 (2 × 10^6^) cells were plated in 100 mm culture dishes and cultured for 48 h. After treatment with various concentrations of aloe-emodin for 48 h, the cells were harvested and washed twice with cold PBS. Protein extracts of cells were prepared by lysing cells in RIPA buffer (Beyotime, Shanghai China) and 1 mM PMSF (Beyotime, Shanghai China) for 30 min at 4 °C. After centrifuged, the protein concentration on supernatant was determined with bicinchoninic acid (BCA) assary (Biomed Beijng China). For each sample, equal amounts of cell lysates (containing 25 μg) were loaded on a 10.0% SDS polyacrylamide gel electrophoresis, and transferred to a PVDF membrane (0.45 μm, BioRad, Cal, USA). Membranes were blocked with blocking buffer (TBST (Beyotime, Shanghai China) and 5% non-fat milk (*w*/*v*)) for 1 h at the RT. Then, the membranes were incubated with primary antibodies overnight at 4 °C. Thereafter, the membranes were washed with TBST buffer and incubated with anti-rabbit secondary antibodies for 1 h at RT. The signals were detected by an Enhanced Chemiluminescence (ECL) system (Tanon, Shanghai, China) according to the manufacturer’s instructions.

### Statistical analysis

All data and results were confirmed by at least three independent experiments and were expressed as mean ± standard deviation (SD). Students’*t*-test was used to analyze cell apotosis, one-way ANOVA followed by Dunnett’s multiple-comparison was used for densitometry analysis of western blots. Calculations were carried out using SPSS version 19.0 and *P* < 0.05 was considered statistically significant.

## Results

### Aloe-emodin reduces viability on SCC15 cell lines

**Fig. 2:**
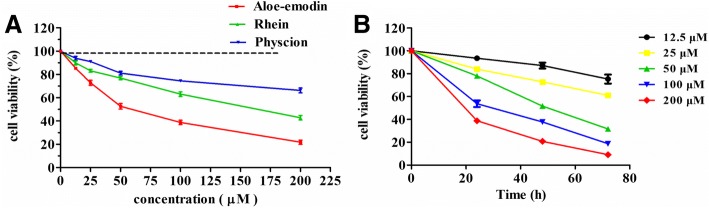
Effects of indicated compounds on the cell viability of the SCC15 cells. **a** Exponentially growing of SCC15 cells was treated with aloe-emodin, rhein and physcion in various concentrations (0, 12.5, 25, 50, 100 and 200 μM) for 48 h. **b** SCC15 cells were treated with 12.5, 25, 50, 100 and 200 μM of aloe-emodin for various times (24 h, 48 h, 72 h). The cell viability was determined using MTT assay. Each bar represents the mean ± SD

The SCC15 cells were treated with various concentrations of aloe-emodin, rhein, physcion respectively. The results showed aloe-emodin, rhein, physcion all inhibited the proliferation of SCC15 cells. The viability of SCC15 cells were reduced to 85.44% with 12.5 μM ranging to 21.79% with 200 μM at 48 h, and the IC_50_ value was 60.90 μM, while the IC_50_ value of rhein was 160.7 μM and physcion was 486.1 μM. The inhibition effects were in the order of aloe-emodin > rhein > physcion (Fig. 2a). The IC_50_ values of rehin and physcion are at high concentrations (> 160 μM) suggesting that the two compounds were of limited value as anti-cancer agents for high dose for oral cancer theaphy. Therefore, aloe-emodin with low IC_50_ value (60.90 μM) was selected for further assessment in SCC15 cells. The number of viable SCC15 cells treated with aloe-emodin decreased in a dose- and time-dependent manner compared to control cells (Fig. 2b).

### Aloe-emodin induces apoptosis in SCC15 cells

**Fig. 3:**
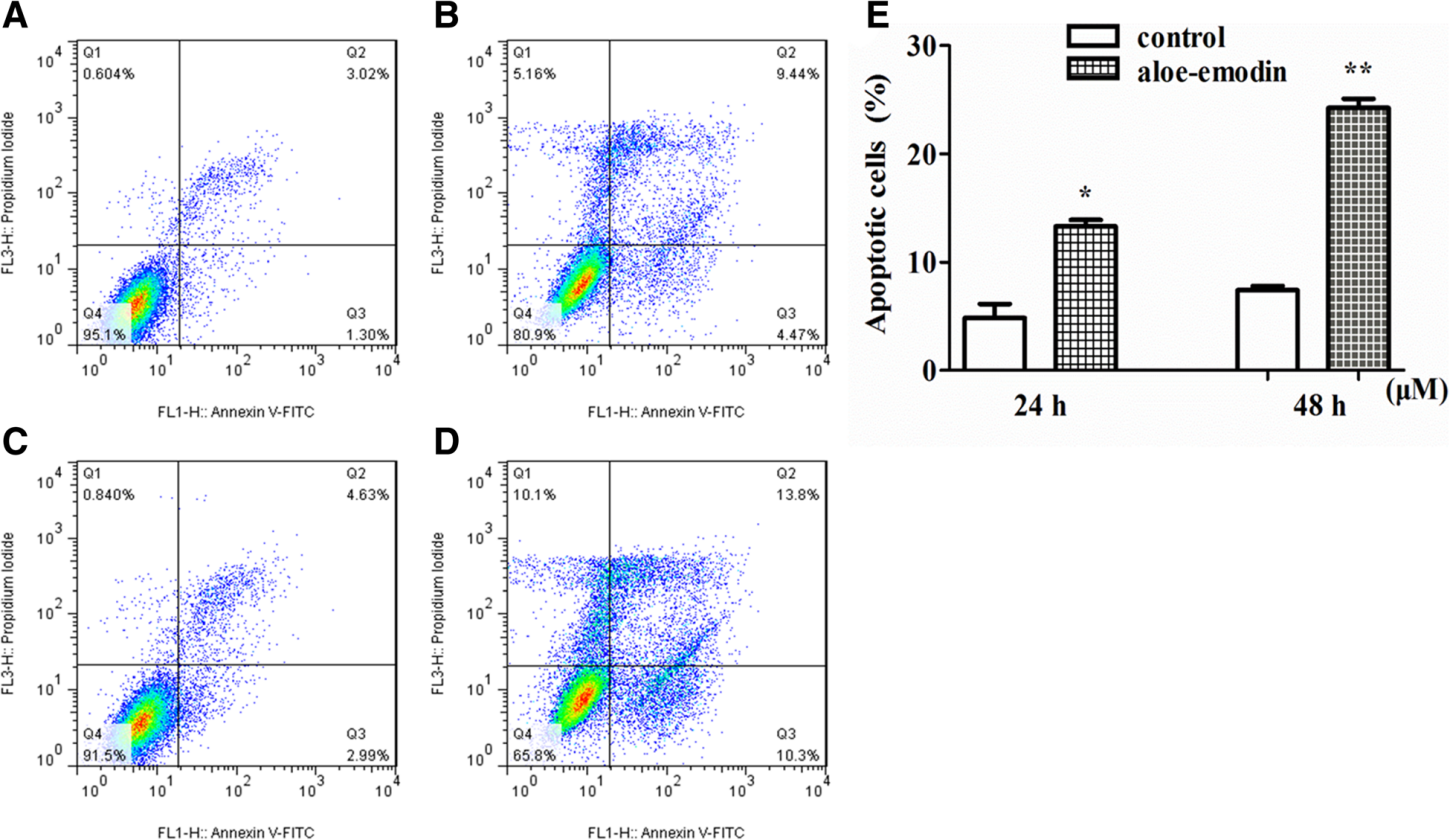
Aloe-emodin induces apoptosis of the SCC15 cells. SCC15 cells were treated by aloe-emodin of 0 μM (**a** and **c**) and 50 uM (**b** and **d**) for 24 h and 48 h, respectively. Annexin binding and propidium iodide (PI) staining were analyzed by FACScan. Q4: viable cells; Q3: early apoptotic cells; Q2: late apoptotic cells; Q1: death cells. The graph visualizes early and late stage apoptotic cells (**e**). Results were expressed as the mean ± SD (*n* = 3) and analyzed by Students’*t*-test (*P < 0.05, **P < 0.01)

To detect whether aloe-emodin induced apoptosis in SCC15 cells, an Annexin V-PI dual staining assay was conducted. The SCC15 cells was treated with 50 μM aloe-emodin for 24 h and 48 h, with the result that apoptotic cell population (early and late stage apoptotic cells) was higher than in the untreated control group. The ratio of apoptosis of SCC15 cells in Aloe-emodin treated group was 13.91% in 24 h and 24.1% in 48 h respectively, while the ration of apoptotic cell was 4.32%, 7.42% in the control group respectively (Fig. 3) (*P < 0.05, **P < 0.01).

### Involvement of caspase-9 and caspase-3 in aloe-emodin induced apotosis

**Fig. 4:**
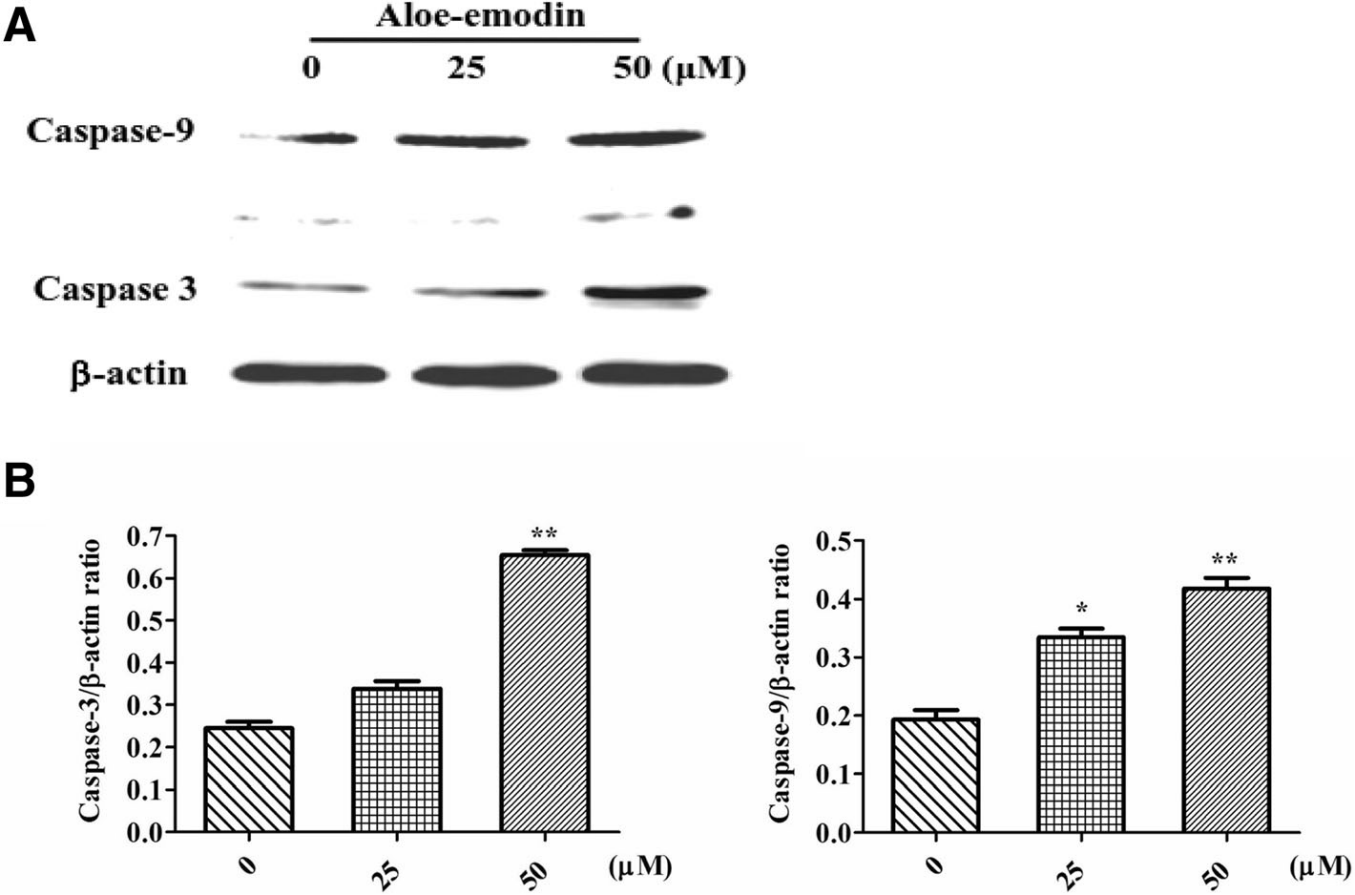
Western blotting analyses of aloe-emodin on the expression of apoptotic proteins. **a** SCC15 cells were treatment with various concentrations (0, 25 and 50 μM) of aloe-emodin for 48 h and the levels of caspase-9, caspase-3 were measured by Western blotting analysis. Increased expression of caspase-9 and caspase-3 were observed. β-actin was used as a loading control. **b** Densitometry analysis of Western blots, Data were expressed as the mean ± SD (*n* = 3) and analyzed by one-way ANOVA using Dunnett’s multiple-comparison test (*P < 0.05, **P < 0.01)

To determine the underlying mechanism by which aloe-emodin induced apoptosis in SCC15 cells, the expression of cell apoptosis molecules caspase-9 and caspase-3 proteins were measured. We found that the expression levels of caspase-9 and caspase-3 increased following aloe-emodin treatment (0, 25 and 50 μM for 48 h) (Fig. 4). These results indicated that aloe-emodin may induces apoptosis by regulating caspase-9 and caspase-3 in SCC15 cells.

## Discussion

Over the past several decades, many studies have reported that natural products derived from medicinal herbs have various potent biological advantages against various types of cancer [9, 10]. Aloe-emodin, rhein and physcion, the anthraquinone anaglogues, are derived from *Rheum undulatum L.* and exhibit anti-inflammatory, anti-bacterial, and anti-tumor properties [11]. Oral squamous cell carcinoma has been reported that the prognosis for patients diagnosed is very poor, less than 50% survive for five years or more and incidence rate is to be younger than other tumors worldwide [12]. Many reports have showed that aloe-emodin, rhein and physcion exhibit anti-proliferative effect and induction of apoptosis in various cancer cells [5, 6, 9]. However, there is no available information to show the effect of aloe-emodin, rhein and physcion against the growth of human oral squamous cell carcinoma SCC15 cells. Herein, we revealed that aloe-emodin, rhein and physcion could exerts anti-proliferative effects on SCC15 cells in vitro, aloe-emodin was selected in further bioactive assessment for the low IC_50_ value, the results demonstrated that aloe-emodin in a time- and dose-dependent decrease in SCC15 cells viability.

Apoptosis plays a critical role in regulating cell death, we detected apoptotic rates using flow cytometry. The apoptotic rate is tested using Annexin V with PI staining. The caspases have been identified to play a vital role in the mechanism of apoptosis [12, 13]. The caspase-3 is considered to be the most important of the executioner caspases, activated caspase-3 can cleave multiple structural and regulatory proteins, that ultimately cause the morphological and biochemical changes seen in apoptotic cells [14]. Caspase-9 is the upstream caspase, the apoptosis process starts with the activation of caspase 9, in turn, activates caspase-3 almost simultaneously, which then activate other caspases, resulting in cell apoptosis. In the present study, we found that the expression levels of caspase-9 and caspase-3 proteins increased, these results may indicate that aloe-emodin induces apoptosis via activation caspase-9 and caspase-3 in SCC15 cells.

## Conclusion

In conclusion, the present study demonstrated that aloe-emodin inhibits the proliferation and induces the apoptosis in SCC15 cells, moreover, we reveal the potential mechanism of apoptosis effect and results indicate that aloe-emodin may be a good entity for anti-oral cancer drug exploring. However, confirmation the results of aloe-emodin against in other OSCC cell lines are necessary and further in vivo studies are required.

## Abbreviations

BCA: Bicinchoninic acid
DMEM: Dulbecco’s modified Eagle’s medium
ECL: Enhanced Chemiluminescence
FBS: Fetal bovine serum
FITC: Fluorescein isothiocyanate
IC_50_: Half maximal inhibitory concentrations
MTT: Thiazolyl blue tetrazolium bromide
OD: Optical density
PBS: Phosphate buffered saline
PI: Propidium iodide
RL: Rheum undulatum L
